# Rising Rates of Obesity Amongst Children on the Autism Spectrum During the COVID-19 Pandemic †

**DOI:** 10.3390/nu17101683

**Published:** 2025-05-15

**Authors:** Wing Yan Yuen, Tammy S. H. Lim, S. V. Karthik, Yijuan Yvonne Lim, Elizabeth M. Teo, Yiong Huak Chan, Liang Shen, Kalyani V. Mulay

**Affiliations:** 1 Department of Paediatrics, Khoo Teck Puat-National University Children’s Medical Institute, National University Hospital, 1E Kent Ridge Road, NUHS Tower Block Level 12, Singapore 119228, Singapore; 2 Child Development Unit, Khoo Teck Puat-National University Children’s Medical Institute, National University Hospital, 1E Kent Ridge Road, NUHS Tower Block Level 12, Singapore 119228, Singapore; 3 Division of Paediatric Gastroenterology, Nutrition, Hepatology and Liver Transplantation, Khoo Teck Puat-National University Children’s Medical Institute, National University Hospital, 1E Kent Ridge Road, NUHS Tower Block Level 12, Singapore 119228, Singapore; 4 Division of Paediatric Endocrinology, Khoo Teck Puat-National University Children’s Medical Institute, National University Hospital, 1E Kent Ridge Road, NUHS Tower Block Level 12, Singapore 119228, Singapore; 5 Yong Loo Lin School of Medicine, National University of Singapore, 1E Kent Ridge Road, NUHS Tower Block Level 10, Singapore 119228, Singapore; 6 Biostatistics Unit, Yong Loo Lin School of Medicine, National University of Singapore, 1E Kent Ridge Road, NUHS Tower Block Level 10, Singapore 119228, Singapore

**Keywords:** pediatric obesity, autistic disorder, COVID-19, overweight, body mass index

## Abstract

*Background:* The COVID-19 pandemic has been associated with rising obesity rates. Autistic children have a higher risk of obesity than neurotypical children. Our study aims to describe the changes in overweight/obesity rates in autistic children during the pandemic, and to identify contributing factors. *Methods:* This is a retrospective case record review of patients with a clinical diagnosis of autism, who were seen at a developmental-behavioral pediatrics clinic in a tertiary academic hospital, between 1 January 2019 and 24 October 2021. We compared the average monthly rates of overweight/obese status pre- and during the pandemic. We collected data on the patients’ and parents’ demographics, duration of screen time per day, degree of difficulties related to autism symptoms and cognition. We analyzed factors associated with being overweight/obese during the pandemic. *Results:* 1330 patient visits were included. The mean age was 45.4 months; 78% were male; 52% were Chinese. The average monthly rate of overweight/obese status increased by 1.8% during the pandemic (17.9% pre-pandemic; 19.7% during pandemic). Factors associated with being overweight/obese during the pandemic included: Malay ethnicity (OR 2.321, *p* < 0.01), developmental delay (OR 2.80, *p* < 0.01), and lower parental education level (father OR 1.73, *p* = 0.01; mother OR 1.63, *p* = 0.03). On multivariate analysis, only Malay ethnicity (OR 2.95, *p* = 0.01) was significant. *Conclusions:* Our study demonstrates a rising overweight/obesity rate amongst children with autism spectrum disorder during the pandemic. It also identified higher-risk patient profiles (Malay race, developmental delay, lower parental education). We hope this will facilitate the implementation of preventative health measures specifically supporting the high-risk children.

## 1. Introduction

The COVID-19 pandemic brought about unprecedented changes in various aspects of daily living for children worldwide. Children on the autism spectrum found it challenging to cope with the changes brought about by the pandemic, resulting in higher levels of psychological distress [1]. Psychological stress and obesity are interconnected through multiple pathways, including cognitive and behavior mechanisms, as well as through physiological and biochemical pathways. For example, stress affects cognitive functions like self-regulation and may increase overeating behaviors [2]. Stress can cause lack of sleep or reduced sleep duration, which are associated with a higher likelihood of obesity [3]. Panchal et al. reported that anxiety and depression symptoms, as well as irritability and anger, were common amongst children and adolescents during the COVID-19 lockdown [1]. Widespread lockdowns and other social distancing measures caused children to have reduced physical activity and increased caloric intake, in what some studies have termed an “obesogenic environment” [4]. Current data from various countries suggest that there has been an increase in obesity rates and Body Mass Index (BMI) in children during the pandemic as compared to the pre-pandemic period [4,5,6,7]. A recent study from Singapore also concluded that cessation of physical activity during the COVID-19 pandemic was associated with increased adiposity in primary school-aged children one year after the pandemic lockdown [7]. Children on the autism spectrum are already known to have a higher risk of obesity than the general population [8]. The disruptions to their lifestyles due to COVID-19 restrictions have made it even more difficult to adhere to healthy lifestyle recommendations [9]. Based on our clinical experience, there is anecdotal suggestion that the overweight and obesity rates in children on the autism spectrum have increased during the pandemic.

Our primary aim is to objectively describe the changes (if any) in overweight and obesity rates in children on the autism spectrum during the pandemic in Singapore. To our knowledge, this is the first study examining this phenomenon in Asian children on the autism spectrum. Our secondary aim is to identify if any factors contributed to the increase in overweight and obesity rates in this cohort. This would facilitate the implementation of specific strategies that would help support the health of this group of children. It is imperative to diagnose and manage childhood obesity as it is strongly associated with adult obesity and its attendant health risks [10].

## 2. Methods

This is a retrospective electronic case record review of patients with a diagnosis of autism, who were seen at a developmental-behavioral pediatrics (DBP) clinic in a tertiary academic hospital between 1 January 2019 and 24 October 2021. This clinic is one of two nationally designated centers in the country assessing and managing preschool children (aged 0 to 6 years old) with developmental, behavioral, and emotional concerns, including autism. We included all in-person patient visits with documented height and weight readings. Patients’ height, weight, and BMI were plotted based on locally normed growth charts. Following the internationally accepted Centers for Disease Control and Prevention (CDC) definition of overweight and obesity, patients who had a BMI between the 85th and 94th percentile were considered “overweight”, while those with a BMI equal to or higher than the 95th percentile were considered “obese” [11]. We also collected data on the patients’ demographics (age, gender, ethnicity), father’s and mother’s age and education level, duration of time spent on the screen per day, degree of difficulties related to autism traits (Social Responsiveness Scale [SRS]) and cognition. The Mullen Scales of Early Learning (MSEL) is a standardized developmental assessment tool routinely used in this center. The Early Learning Composite (ELC) score is used to provide an overall estimate of the child’s cognition. A score of less than 85 signifies 1 standard deviation below the mean (presence of overall reduced cognitive functioning). The study was approved by the institution’s ethics review board. A waiver of informed consent was approved as this was a retrospective medical record review.

We compared the average monthly rates of overweight or obese status during the study period. All our patients follow standard review frequencies in our clinic. Hence, we used a time-based approach to analyze anthropometric data that would give us a good aggregate trend of our patients’ overweight/obesity rates. The pre-pandemic period was defined as January 2019 to January 2020 and the pandemic period from February 2020 to October 2021. The pandemic period was defined as such because the country’s national pandemic alert level, known as the Disease Outbreak Response System Condition (DORSCON) level, was escalated to Orange (2nd highest level) on 7 February 2020 [12]. We further analyzed factors that were associated with being overweight or obese during the pandemic.

Data were analyzed using the IBM SPSS Version 26. Categorical data were reported as percentages. Continuous variables were expressed as means with standard deviation (SD). Univariate and multivariate analyses were conducted for the factors associated with overweight or obesity, with results expressed using odds ratio (OR) and a 95% confidence interval (CI).

## 3. Results

A total of 1330 eligible patient visits were analyzed during the study period of 1 January 2019 to 24 October 2021, of which there were 285 visits in the pre-pandemic and 1045 visits in the pandemic period. The mean age of the patients was 45.4 months (SD 17.7 months) and 78% of them were male. A total of 52.1% of the patients were of Chinese ethnicity, 23.4% were of Malay ethnicity, 10.5% were of Indian ethnicity, and 13.9% identified as “Others”. According to Singapore’s population census in 2020, Chinese, Malays, and Indians constituted 74.3%, 13.5%, and 9.0% of the resident population respectively [13].

**Figure 1:**
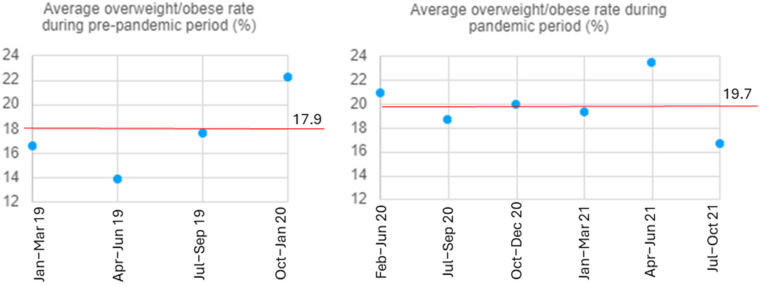
Average overweight/obese rate during pre-pandemic and pandemic periods.

As shown in Table 1 and Figure 1, the average monthly rate of overweight or obese children on the autism spectrum increased by 1.8%, from 17.9% pre-pandemic to 19.7% during the pandemic. The change was more pronounced in certain groups: children aged four years old or below (increase of 4.5%), non-Chinese children (increase of 4.6%), children whose parents’ education level were post-secondary school or below (increase of 5.0%), and children with screen time of more than 2 h per day (increase of 5.7%).

**Figure 2:**
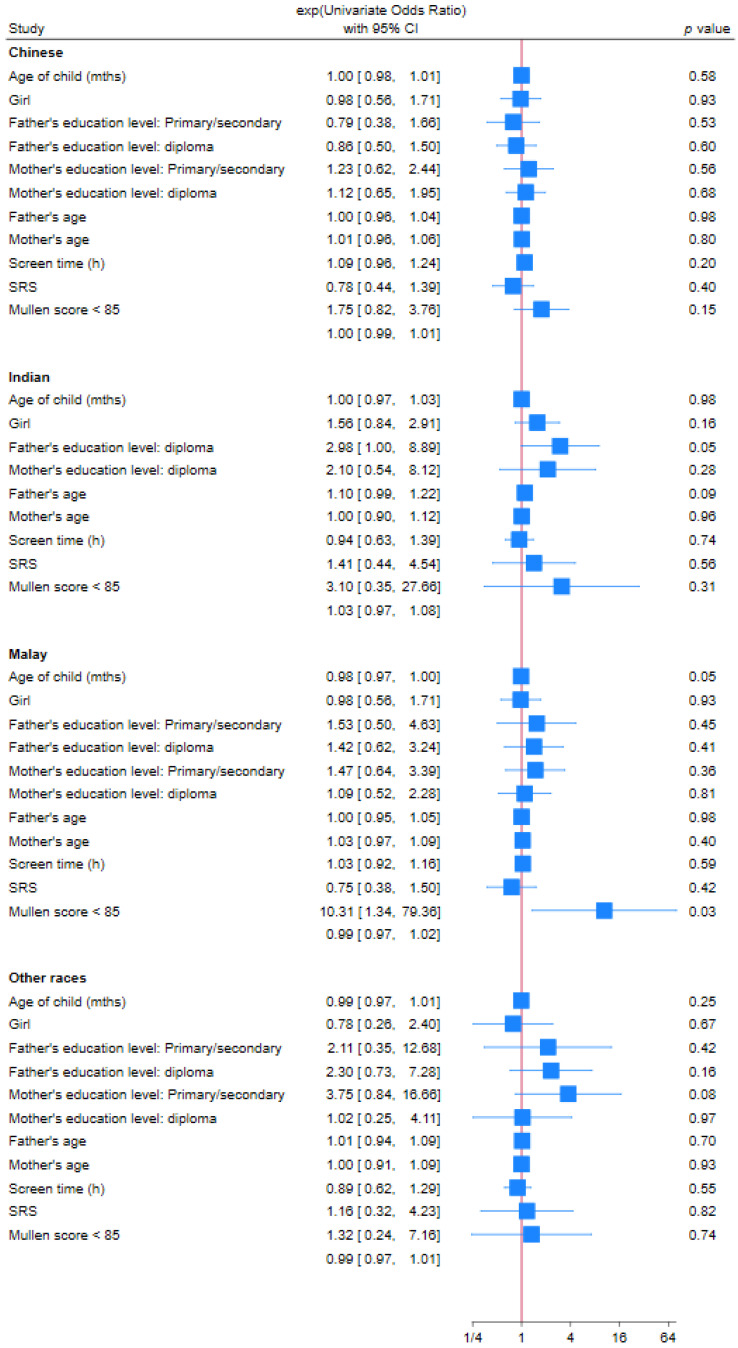
Comparison of demographic variables according to ethnicity. Note: Not possible to have estimates for father’s/mother’s educational level primary/secondary in the Indian population due to small group size.

Factors associated with being overweight or obese during the pandemic in children on the autism spectrum were analyzed as shown in Table 2. Univariately, risk predictors of overweight or obesity were: Malay ethnicity (OR = 2.32, 95% CI 1.64–3.28, *p* < 0.01), fathers with post-secondary or diploma highest education level (OR = 1.56, 95% CI 1.10–2.20, *p* = 0.01), mothers with primary or secondary highest education level (OR = 1.63, 95% CI 1.05–2.55, *p* = 0.03), and MSEL ELC score less than 85 (OR = 2.80, 95% CI 1.53–5.15, *p* < 0.01); older age of child (OR = 0.99, 95% CI 0.981–0.998, *p* = 0.02) was a protective factor. Upon multivariate logistic regression, only Malay ethnicity remained a significant risk factor (OR = 3.29, 95% CI 1.39–7.74, *p* = 0.01). Table 3 shows the detailed demographic data for these variables across different ethnic groups. There are statistically significant differences in the ethnic distribution across all the other variables being examined. Compared to the ethnic distribution of our study cohort, there is a higher proportion of parents of Malay ethnicity having a lower education level, as well as a higher proportion of Malay children who are younger and having lower MSEL scores. We further performed subgroup analysis of the risk factors by ethnic group, as shown in the forest plot in Figure 2. Only within the Malay population, a lower MSEL score is a risk factor for obesity (OR = 10.3, 95% CI 1.34–79.36, *p* = 0.025), while older age is a protective factor against obesity (OR = 0.98, 95% CI 0.97–1.00, *p* = 0.046).

## 4. Discussion

After the onset of the COVID-19 pandemic, a new term “covibesity” was coined, suggesting that the pandemic has had a detrimental effect on obesity [14]. This is supported by studies all over the world, which show an increase in obesity rates and BMI in neurotypical children during the pandemic, as shown in Table 4. Dong et al. in China found that the prevalence of overweight and obesity increased from 9.2% and 8.6% before the pandemic, to 10.5% and 10.6% during the pandemic, respectively [6]. Similarly, data from the US showed that on average, overall obesity prevalence increased from 13.7% before the pandemic to 15.4% during the pandemic [5]. In another longitudinal cohort of persons aged 2–19 years with outpatient visits, the monthly rate of increase in BMI nearly doubled during the COVID-19 pandemic, compared to the pre-pandemic period [15]. These studies on obesity are not specific to children on the autism spectrum, and most of the current studies published on autistic children during the pandemic have focused on the psychological impact of COVID-19 instead. Our study filled this gap in the literature and demonstrated a comparable increase (1.8%) in the rate of overweight or obese children on the autism spectrum during the pandemic, as compared to the increase in rates of 1.3% to 3% reported by studies on neurotypical children. Local data comparing the proportion of school-going children (not specific to those with autism) who were overweight from 2017 to 2021, showed an increase from 13 to 16%, especially from 2020 to 2021, during the pandemic [16]. In our cohort, the baseline overweight/obesity rate of 17.9% pre-pandemic is higher than that in a more general cohort of children, but consistent with rates reported in the literature for children on the autism spectrum [17]. A systematic review by Sammels et al. also showed that autistic children have a higher risk of obesity compared to neurotypical peers [17]. However, our study did not demonstrate a higher increment in obesity rates in autistic children, compared to a general cohort of children during the pandemic. Further studies involving a larger cohort of children with a matched comparison group of neurotypical children would give us information on this aspect. Whilst longer-term data on whether the high obesity rates persisted post pandemic are not yet available, the known natural history of obese children is that the majority grow up to be obese adults [10]. While the increase of 1.8% in the obesity rate in our study population may seem marginal, this increase was observed over a period of merely 2 years, and highlights the impact of the pandemic on accelerating the obesity rate within a short period of time. Thus, it is important to detect childhood obesity early and intervene to reduce the risk of the child becoming an obese adult as this is associated with adverse health consequences and high healthcare costs to society.

In Singapore, the lockdown, also known as the circuit breaker, was introduced from 7 April to 1 June 2020, 16 May to 13 June 2021, and 22 July to 9 August 2021. All public places including schools, playgrounds, and parks were closed during the above-mentioned periods. The increase in obesity rates and BMI can be explained by the effect of such measures taken to reduce the spread of COVID-19, as it results in reduced physical activity, increased screen time, and increased consumption of processed or calorie-dense foods during the period [18]. For some children whose main or even only physical activity is during the mandated physical education lessons in schools, school closure can result in their lifestyle becoming completely sedentary. Individuals on the autism spectrum may possess reduced motivation for physical activity [19]. As such, this group is understandably at a higher risk of overweight and obesity than the general population. The pandemic is also thought to have impacted on the eating behaviors of children on the autism spectrum, with an increased frequency of consumption of high-calorie foods, such as sweets [20].

In our study, we note the differences in the ethnic group distribution in our study population (52.1% of the patients Chinese, 23.4% Malay, 10.5% Indian, and 13.9% identified as “Others”) as compared to the Singapore population census (74.3% Chinese, 13.5% Malay, 9.0% Indian, 3.2% Others). As part of a tertiary institution, our unit accepts international patient referrals (with ethnicities other than Chinese, Malay, and Indian), in addition to local patients. This may explain why the proportion of “Others” ethnicity is higher in our study population. Our unit also provides government-subsidized services predominantly (as opposed to self-funded private services). Hence, we see a higher proportion of patients of Malay ethnicity compared to population norms, as the Malay ethnicity has a lower reported median household income compared to other ethnic groups. Patients from other ethnicities (Chinese/Indian) with higher median household incomes may be accessing private care outside of our institution.

We found that Malay ethnicity was significantly associated with increased odds of being overweight or obese during the pandemic. Local adult data, even before the pandemic, consistently reports that the Malay ethnic group has a higher rate of obesity, compared to the other groups [21]. Similar data for children are currently unavailable. However, there are pre-pandemic data to suggest that the diet quality of Malay children in Singapore is lower compared to other ethnic groups. Brownlee et al. evaluated the diet of a sample of Singaporean children using a scoring system based on the compliance to food-based dietary guidelines by the Singapore Health Promotion Board. They found that total diet quality scores for Malay children were statistically lower compared to other ethnic groups. Malay children also consumed fewer fruits, vegetables, and dairy, but more sodium and added sugars, compared to other ethnic groups; such eating patterns may put the Malay children at higher risk of overweight/obesity at the baseline [22]. In Singapore, the majority (98.8%) of Malays adopt a Muslim diet, necessitating that foods consumed have Halal certification [23]. This may limit the range of healthy foods available to this group locally. Although our study did not collect information on parents’ income, national per capita income data reports for Singapore show lower incomes in Malay compared to other ethnic groups [24]. It is thought that there may be limitations in the availability of culturally appropriate healthy and affordable food options in this ethnic group. Extant literature also demonstrates the ethnic disparities in rates of obesity for children on the autism spectrum, mediated by food insecurity [22]. Moreover, data from the Singapore Longitudinal Early Development Study also showed that the COVID-19 pandemic disproportionately exacerbated socioeconomic inequalities for children, including food insecurity, especially in those with a lower socio-economic status [25]. Tester et al. suggested that for families with limited purchasing power, the “per calorie” cost of nutrient-dense fresh foods is higher than that for calorie-dense processed foods, i.e., processed food requires a lower cost for satiety at the expense of a higher obesity risk in families of lower socioeconomic status [26]. In our study, the Malay parents had a lower education level compared to the rest of the ethnic groups, consistent with local population data. Locally, there are discrepancies in the education level across the different ethnic groups. The proportions of Singapore residents with polytechnic or university qualification are 31% in Indians, 27% in Chinese, and 9% in Malays [27]. This difference in the parental education level may be another driving factor resulting in the Malay ethnic group having an overall higher risk of obesity, as a lower parental education level is a known risk factor in childhood obesity [28]. This knowledge is crucial for developing nuanced healthcare policies that consider the specific risk profiles and concerns of each ethnic group, for successful health promotion for all children.

In our analysis, the father’s or mother’s education level of degree and above was used as the reference category, as the average parent education level in this cohort was a degree. The increase in the average monthly rate of overweight or obese children on the autism spectrum was higher in children whose parents had lower education levels. In a study of parents’ knowledge, attitudes, and practices on childhood obesity in Singapore, it was found that lower-educated parents are more likely to report negative feedback on health knowledge and health practices. In turn, their children are at a higher risk of being obese as compared to children of parents who have positive feedback on health knowledge and practices [29]. Studies carried out during the pandemic in neurotypical children also showed that a higher level of parental education was associated with less weight gain in children during the early phase of the pandemic, as well as a negative association with a BMI z-score change [30,31]. This is possibly contributed by a difference in the health behaviors between families, as a Spanish study found that a worsening of health habits was also found to be more accentuated in families with low educational levels during the pandemic [32].

With the lockdown, it would be difficult to keep children engaged at home and screen time use was higher in children during the pandemic compared to pre-pandemic [33]. We found that the increase in the average monthly rate of overweight or obese was higher in children on the autism spectrum with screen time of more than 2 h a day, although this was not statistically significant. It is possible that our study was not adequately powered to detect a statistically significant relationship between higher screen time per day and increased overweight or obesity rates in this cohort. At the baseline, screen time per day in autistic children is higher than that seen in typically developing children; this remained so during the pandemic as total screen time per day increased in both populations [34]. This trend was further exacerbated in countries with a longer duration of lockdown, with poorer dietary habits being associated with increased screen time [35]. The pandemic-related increased screen time per day also tended to persist even after public health measures were lifted, suggesting that its impact on diet, displacement of physical activities and weight is likely to persist beyond the pandemic [36].

In our study, we also found that having lower cognitive functioning is associated with increased odds of being overweight or obese during the pandemic. This is consistent with the literature in that lower cognitive functioning is reported to be associated with higher BMI in childhood, as these children tend to have poorer health behaviors, such as unhealthy dietary habits and less physical activity [37].

The onset of obesity is usually in later childhood or adolescence [38]. However, our analysis revealed that the older age of the child is a protective factor against obesity in our cohort during the pandemic. In a recent study by Jenssen et al., which examined the changes in obesity trends in children during the pandemic, they found that the increase in obesity rates was more marked for younger patients aged 5 to 9 years (2.6%) as compared to an increase of 1.0% for patients aged 13 to 17 years [5]. Another study by Shalitin et al. in Israel also found increased weight gain in younger children (aged 2–6 years old), compared to older children (aged 6–18 years old) [39]. In a Swedish study involving preschool children, increased rates of overweight and obesity were observed in children aged 3–4 years old [40]. None of these studies looked specifically at children on the autism spectrum. In our cohort, the differences in overweight or obesity trends could already be appreciated at a younger cut-off age of four years, possibly due to the higher risk of overweight or obesity in children on the autism spectrum. This is a worrying trend as it means that interventions should be conducted at an early stage before the child’s feeding preferences and habits become entrenched [41]. Younger children on the autism spectrum may have more behavioral challenges, as compared to those who are older [42]. Parents may resort to offering more food rewards (typically high-calorie processed food items or candy) and screen time as strategies to keep the children occupied or in an attempt to resolve tantrums. These strategies may explain the increase in weight status in this group of children. The majority (88–95%) of preschool-aged children attend childcare in Singapore and these children eat at least one–two meals in school [43]. Schools are required to follow local Healthy Meals in Preschools Program guidelines per the Early Childhood Development Centers Regulatory Standards for provision of healthy, balanced meals [44]. As such, pandemic-related school closures for younger children can result in the loss of nutritious meals, and parents working from home while caring for the children may struggle to provide meals of similar quality at home [45]. Many young children, especially those on the autism spectrum, experience feeding difficulties and food selectivity, including preference for high-calorie processed foods, which puts the child at increased risk of obesity [46]. In contrast, older children may be less susceptible to this effect as feeding difficulties may have a trend towards an age-related improvement [47]. In our experience, many of our patients above the age of four years would have been referred to specialist feeding services and have received feeding intervention to improve their feeding difficulties.

Our study is the first study in the world describing changes in overweight and obesity rates in children on the autism spectrum during the COVID-19 pandemic in the Asian population. In addition, this study provides preliminary data on certain profiles (e.g., Malay ethnicity, lower parental education level, younger age, lower cognition, higher screen time usage) that are at a higher risk of being overweight or obese. With this information, specific interventions that could be implemented during clinic visits would include detailed history-taking for diet, screen use, and physical activity in all patients, as well as a higher frequency of monitoring anthropometry and a lower threshold of referral to the pediatric endocrinologist and dietitian for weight management in patients at a higher risk of being overweight or obese. Specific to patients of Malay ethnicity, clinicians may also provide healthy eating resources that are culturally appropriate, including lists of healthier snacks that are Halal-certified as the majority of Malays in Singapore are Muslim and effort is needed to read food labels to determine if the snacks are in line with their religious beliefs.

We acknowledge that our study has its limitations. Firstly, the retrospective study design does not allow causality to be examined and presents risk of bias due to missing or poorly recorded data. In addition, we do not have adequate documented data to examine other important variables associated with childhood obesity, such as caloric intake, physical activity, income level of the family, and family history of obesity. While self-reported data on screen time may also be inaccurate or underestimated, in the absence of more sophisticated methods of collection of screen time data that can be obtained in a prospectively designed study, this was the best compromise in our study. In future, our team can consider obtaining such information for routine clinical practice, especially in children who are at higher risk of overweight/obesity as well as counsel against excessive screen time during meals. This study looked at obesity rates pre-pandemic and during the pandemic, and did not evaluate whether there were lasting effects or recovery post pandemic, which would be important for future research. Lastly, this study was conducted in a single institution, which typically may limit the generalizability of our findings. However, as our institution is one of only two public institutions in Singapore serving children on the autism spectrum disorder, we receive a high clinical load of such patients regularly. Therefore, we believe that our findings can be generalized to Singaporean children on the autism spectrum. We acknowledge that these findings may be specific to Singapore and may not necessarily be generalizable to autistic children in other parts of the world. More studies may need to be conducted in other cohorts of autistic children.

## 5. Conclusions

Our study suggests that there was an increase in the average monthly rate of overweight or obese children on the autism spectrum attending our clinic during the COVID-19 pandemic, and Malay ethnicity was significantly associated with increased odds of being overweight or obese. The study underpins the need to explore barriers to accessing healthy lifestyles for specific groups of children and their families. Preventive health measures should be tailored to address the issue of obesity for children on the autism spectrum with special focus on ethnic minority groups and households with parents of lower educational levels.

## Figures and Tables

**Table 1:** Average monthly rates of overweight or obese children on the autism spectrum.

	Pre-Pandemic ^†^ Rate, %	Pandemic ^§^ rate, %	Difference in Rate, %
Overall (*n* = 1330)	17.9	19.7	1.8
Child’s age			
≤4 years	17.3	21.7	4.5
>4 years	21.5	18.0	−3.5
Ethnicity			
Chinese	14.5	15.3	0.8
Non-Chinese	21.3	26.0	4.6
Parent’s highest education level			
Diploma and below	16.9	22.0	5.0
Degree and above	18.1	19.4	1.3
Screen time			
>2 h	15.3	21.0	5.7
≤2 h	22.5	24.1	1.6

^†^ Pre-pandemic period defined as January 2019 to January 2020. ^§^ Pandemic period defined as February 2020 to October 2021.

**Table 2:** Risk factor analysis for being overweight or obese during the pandemic in children on the autism spectrum.

	Univariate OR (95% CI)	*p* Value	Multivariate OR (95% CI)	*p* Value
Age of child	0.99 (0.981–0.998)	**0.02**	1.01 (0.98–1.03)	0.54
Gender (Female)	1.07 (0.74–1.53)	0.73	1.14 (0.50–2.61)	0.76
Race				
Chinese	Reference			
Malay	2.32 (1.64–3.28)	**<0.01**	3.29 (1.37–7.74)	**0.01**
Indian	1.20 (0.70–2.09)	0.5	2.90 (0.90–9.32)	0.07
Others	1.43 (0.90–2.26)	0.13	0.26 (0.03–2.07)	0.2
Father’s education level				
Degree and above	Reference			
Post-Secondary or Diploma	1.56 (1.10–2.20)	**0.01**	1.71 (0.51–5.71)	0.38
Primary or Secondary	1.07 (0.62–1.82)	0.81	0.82 (0.34–1.98)	0.66
Mother’s education level				
Degree and above	Reference			
Post-Secondary or Diploma	1.35 (0.94–1.94)	0.1	0.44 (0.13–1.48)	0.19
Primary or Secondary	1.63 (1.05–2.55)	**0.03**	0.97 (0.42–2.26)	0.95
Father’s age (years)	1.00 (0.97–1.02)	0.68	1.01 (0.94–1.08)	0.78
Mother’s age (years)	1.00 (0.96–1.02)	0.61	0.98 (0.90–1.08)	0.73
Screen time > 2 h/day	1.07 (0.99–1.16)	0.1	1.05 (0.93–1.18)	0.45
SRS T-score ≥ 60	0.82 (0.56–1.21)	0.32	0.79 (0.38–1.67)	0.55
MSEL ELC score < 85	2.80 (1.53–5.15)	**<0.01**	0.99 (0.41–2.44)	0.99

MSEL ELC: Mullen Scales of Early Learning Early Learning Composite; SRS: Social Responsiveness Scale.

**Table 3:** Detailed demographic data across ethnic groups.

Significant Variables on Univariate Analysis	Ethnic Distribution (%)			
	Chinese	Malay	Indian	Others
Age of child (*p* = 0.006)				
4 years and below	50%	26.6%	10.2%	12.4%
Above 4 years	54.1%	18.7%	11.0%	16.1%
Father’s education level (*p* < 0.001)				
Degree and above	59.5%	8.0%	14.5%	18.1%
Post-Secondary or Diploma	43.8%	43.8%	6.9%	5.4%
Primary or Secondary	62.0%	27.5%	4.2%	6.3%
Mother’s education level (*p* < 0.001)				
Degree and above	56.8%	10.1%	15.6%	17.6%
Post-Secondary or Diploma	50.3%	39.5%	4.8%	5.4%
Primary or Secondary	51.2%	37.8%	5.2%	5.8%
MSEL ELC Score (*p* < 0.001)				
<85	50.9%	29.6%	9.2%	10.3%
≥85	70.1%	15.3%	8.3%	6.3%
Reference: whole study cohort	52.1%	23.4%	10.5%	13.9%

MSEL ELC: Mullen Scales of Early Learning Early Learning Composite.

**Table 4:** Comparison of rates of overweight or obese children during the pandemic worldwide.

Study/Report	Weight Category	Pre-Pandemic Rate, %	Pandemic Rate, %	Difference in Rate, %
Current Study	Overweight or obese	17.9	19.7	1.8
Ministry of Health, Singapore [16]	Overweight	13	16	3
Dong et al. [6]	Overweight Obese	9.2 8.6	10.5 10.6	1.3 2.0
Jenssen et al. [5]	Obese	13.7	15.4	1.7

## Data Availability

The original contributions presented in this study are included in the article. Further inquiries can be directed to the corresponding author.
